# Implementation of cloud computing in the German healthcare system

**DOI:** 10.1038/s41746-024-01000-3

**Published:** 2024-01-13

**Authors:** M. Putzier, T. Khakzad, M. Dreischarf, S. Thun, F. Trautwein, N. Taheri

**Affiliations:** 1 Center for Musculoskeletal Surgery, Charité – Universitätsmedizin Berlin, corporate member of Freie Universität Berlin, Humboldt-Universität zu Berlin, Berlin, Germany; 2 RAYLYTIC GmBH, Petersstraße 32 – 34, 04109 Leipzig, Germany; 3 Core Facility Digital Medicine and Interoperability, Berlin Institute of Health at Charité–Universitätsmedizin Berlin, Berlin, Germany; 4 Berlin Institute of Health, Julius Wolff Institute for Biomechanics and Musculoskeletal Regeneration, Charité-Universitätsmedizin Berlin, Augustenburger Pl. 1, 13353 Berlin, Germany

**Keywords:** Health policy, Health care economics, Health services

## Abstract

With the advent of artificial intelligence and Big Data - projects, the necessity for a transition from analog medicine to modern-day solutions such as cloud computing becomes unavoidable. Even though this need is now common knowledge, the process is not always easy to start. Legislative changes, for example at the level of the European Union, are helping the respective healthcare systems to take the necessary steps. This article provides an overview of how a German university hospital is dealing with European data protection laws on the integration of cloud computing into everyday clinical practice. By describing our model approach, we aim to identify opportunities and possible pitfalls to sustainably influence digitization in Germany.

## Introduction

The need for digitization in the German healthcare system is undisputed. But in a globalized world, where various sectors of the economy can internationally interact without problems, the innovation and modification of healthcare systems specifically in Germany, have proven to be a difficult undertaking^1^.

The hurdles of digitization are ethically perfectly understandable and justifiable: physicians do not work with economic data, but more importantly with sensitive confidential information. The mishandling and possible privacy breach of secret patient data potentially poses serious real-world consequences for the patient^2^. The protection of the privacy of patient data and their confidential information therefore is the logically overriding maxim in the implementation of digital ecosystems in hospitals - especially when aiming for the goal of establishing paperless hospitals^3^. While innovation in the German healthcare system has picked up speed in recent years, there is room for improvement in a global comparison of the Electronic Medical Records Adoption Model (EMRAM) score^4^. The EMRAM Score is a tool to describe the level of digitization in hospitals on 7 levels with level 1 representing the lowest and level 7 the highest degree of digitization.

The need for digitized medicine does not only result from logistical, economic and ecological advantages, e.g. reduced storage space, less paper waste and savings in maintenance costs, but also leads to improved and more individual diagnostic and therapeutic concepts through the merger of local, regional and national hospitals into digital medical ecosystems. Furthermore, digitized medicine can enable more precise outcome analysis through improved and coherent longitudinal tracking of patient outcomes and provide feedback for therapeutic decisions. In the context of paper-based records, such data is either not captured at all or is lost over time^5^.

The creation of a digital medical ecosystem can thus establish better therapy algorithms and ensure significantly better treatment options on the premise of sharing medical data and the establishment of Big Data sets^6^. The sheer flood of highly scaled and information-dense data requires innovative technologies that further improve data sharing in medicine as it is almost fully accepted as standard and will develop further in the future^5^.

With the importance of the topic, it is understandable that digitization, digital ecosystem and cloud computing are buzzwords. In 2008, only 2 articles were published considering the above-mentioned topics, since then the number grown significantly with 820 articles considering cloud computing had been published in 2022 alone.

Several review articles address the theory of technical regulations and how to maintain adequate data integrity, confidentiality, anonymity and authenticity, only a few have given recommendations how to actually integrate a cloud system in a working clinic^7–9^. While in theory, a conversion to digital ecosystems is not really an issue, one has to face the reality that a complete conversion is a rather complex process^10^.

Therefore, we would like to present you the approach that we have successfully established at the Charité – University Hospital Berlin. Currently, there is no German hospital fully running on cloud computing. The approach described within this article characterizes a model approach to implementation of cloud computing within the framework of a running hospital information system (HIS). As this model approach is characterized by easy accessibility, HL7v2 – and FHIR interoperability as well as the possibility of using it without total integration into the current HIS, we aim to provide an example for other hospitals.

### Cloud computing

Digital ecosystems are generally run on clouds. “Cloud computing” refers to the paradigm of delivering computational resources, including storage, processing power, and applications, as on-demand services over the internet. This model enables users to access and utilize these resources without the need for upfront infrastructure investments, allowing for flexible scalability and cost efficiency. Therefore, saving upfront investments for connection, individualized interfaces that cater to hospital’s needs and are able to adapt to new developments. Cloud computing is characterized by its service models, namely Infrastructure as a Sevice (IaaS), Platform as a Service (PaaS), and Software as a Service (SaaS), which offer varying levels of control and management for users. This technology has gained significant traction due to its potential to revolutionize IT infrastructures and support various industries, such as healthcare, finance, and entertainment. Notably, cloud computing has been acknowledged for its role in facilitating resource sharing, improving accessibility and enabling collaboration among geographically dispersed users^11,12^.

Currently, cloud computing is being used successfully in various areas of medicine: In the provision and processization of telemedicine services^13^, medical image analysis both for oncology services^14^ and preoperative planning e.g. for hip arthroplasties^15^, and in the context of citizen health applications to process lifestyle-related data and recommend lifestyle changes^16,17^. Cloud computing finds therapeutic use in supporting treatment decisions^18^, early sepsis detection, and computation of complex procedures such as Montecarlo simulations for radiotherapy^19,20^. Furthermore, there are already some examples of the implementation of cloud computing as a clinical operating system: In China, for example, large regional hospitals exchange data about patients in a cloud with small grassroot hospitals. The usage of a cloud as SaaS leads to an investment reduction of around 90% while establishing sufficient and modern digital infrastructure^21^.

The predecessor of avant-garde cloud computing are HIS are often described as legacy systems or legacy interfaces referring to computing software that has been outdated by recent technological advances. While legacy interfaces still meet the needs they were designed for (e.g., assessment of personal data, connection between different clinical specialties), they are costly to maintain, use up both computational and physical space and make innovation of their systems difficult. A simple switch to cloud computing could overburden legacy system manufacturers organizationally and financially, so a step-by-step migratory or integrative approach is preferable.

In comparison: cloud computing enables ubiquitous, convenient, on-demand network access to a shared pool of configurable computing resources (e.g., networks, servers, storage, applications, and services). These resources are accessible with minimal effort or extensive provider interaction^22^.

Currently, no complete cloud HIS software solution has been implemented in Germany. However, 98% of healthcare organizations are already running at least one of their applications in a cloud^23^. The global adoption of the FHIR standard is spearheaded in Germany through projects like the Medical Informatics Initiative, the Berlin Institute of Health (BIH) Health Data Platform or the AIQNET project, laying the foundation for the interoperability of medical data. The consensus along experts - not only from Germany - points out that the healthcare system is more than ready for the start of implementation of cloud-based medical data applications, which have the potential to create a decentralized, interoperable ecosystem for the legitimate use and exchange of medical data^24^. The use and improvement of Cloud Computing is mainly driven by the advantages of resilience, networking, and strict adherence to data protection^25^. The technical advantages of cloud computing cannot be denied. Apart from various technical challenges, which primarily occupy software developers, the implementation in everyday clinical practice is primarily dependent on the preservation of data protection and medical confidentiality in the processing, transfer, storage and retrieval of sensitive data^26–28^.

### Legal requirements for cloud computing in Germany and the EU according to GDPR

While healthcare remains to be a nationally regulated matter, the EU’s limited competences regarding the health care system do not apply to data protection competences^29^. EU countries and therefore also German hospitals are subject to the EU’s General Data Protection Regulation (GDPR)^29^. The GDPR regulates the processing of personal data by or on behalf of hospitals. According to GDPR, the processing and storage of personal data can either be a fully, partially or not at all automated process. This includes processes within clinical interfaces and the tasks they are supposed to fulfill^30^.

Based on Art.28 of the GDPR, the use of an external service provider (e.g., cloud computing providers) bound by instructions for the processing of personal data is possible under the general premise that data is not forwarded to third parties. From a legal point of view, this describes commissioned processing; the supervising client – meaning the person or company that has transferred the processing of the data to a cloud provider - remains responsible for the data and its security. According to Art. 4(7), 4(8) and 4(10) of the GDPR, the processor is not a third party, rather the processing is attributed to the controller^30^. The controller is defined as being a person, company or other organization responsible for determining how personal data is used. It is of upmost importance to clearly define the roles of every involved party.

Furthermore, the GDPR requires the precise treatment of the subject’s data and duration of the processing, the nature and purpose of the processing, the type of data, categories of data subjects, and the obligations and rights of the controller. According to Art. 28(3) of the GDPR, processing of personal data may only be carried out on instruction and by authorized persons. The third-party provider must consult the responsible person regarding technical infrastructure, compliance with obligations and the handling of any data protection breaches (Art. 33, 34 GDPR), apart from technical and organizational standards^31^. This means that both the clinic as well as the company supplying the cloud computing SaaS have to legally define the handling of the subject’s data and above-mentioned criteria of processing.

Medical confidentiality in Germany is determined by medical professional law (§ 9 MBO-Ä), the treatment contract (§§ 630a-630h BGB) and criminal law. Every violation of it is considered a criminal offense. Violations describe unauthorized disclosures of other people’s confidential information (from the personal sphere, business or trade secrets) entrusted to him as a member of a medical profession. Violations of confidentiality are punishable by fines or imprisonment^32^.

Considering potential collaborations with external service providers that operate cloud computing in third countries is accurately as well as restrictively regulated by the GDPR. In order for a European or German hospital to be able to cooperate with an external service provider with its headquarters in the USA, a sufficient level of data protection must be guaranteed in accordance with Art. 44 of the GDPR^33^. Such adequacy decisions have been described for the following countries: Andorra, Argentina, the Faroe Islands, Guernsey, Israel, the Isle of Man, Japan, Jersey, New Zealand, Switzerland, Uruguay and, to a limited extent, Canada. Since the 10^th^ of July of 2023 the European Commission has adopted its adequacy decision for the EU-U.S. Data Privacy Framework. Therefore, data transferred between the countries is currently considered as protected as it is in the EU. However, the Austrian data privacy activist Max Schrems, who brought the previous two US-EU regulations to a fall, sees the current data privacy regulations nearly unchanged from the previous versions. It remains to be seen whether the current regulations will stand up to review by the EU court of justice, so there is still uncertainty regarding the use of data processors based in the USA.

### Legal requirements for cloud computing in Germany according to German federal law, state law and hospital law

Since 2017, according to §203(3) para. 4 of the StGB (German Criminal Code), it is possible to involve external service providers for assistance activities for health care professions, which are described under §203 STGB^33^. After the reform, cloud computing providers have been regarded as aiding and abetting the persons responsible for the secrecy of judgments. This development should be rated as a sign from the federal government that the necessity of the development towards cloud computing must also be simplified in the legislative level. The reduction in the protection of secrets is compensated for by the inclusion of the external service provider in the criminal liability for violations. Furthermore, the Confidentiality Reform Act^34^ describes that the economic advantage of storing data on an external information technology system (cloud) may be used if data protection is complied with.

The law also stipulates that the client and external service providers must ensure that the latest technical and organizational measures are in place to prevent the leak of personal data. Possible measures include anonymization, pseudonymization or data encryption using a key from the confidentiality provider^35^.

Furthermore, the federal division of Germany poses further difficulties for the implementation of digital medical ecosystem as the federal states own hospital laws can be restrictive to varying degrees: For example, in Berlin, the Federal Data Protection Act (BDSG)^36^ applies to all hospitals in public or rural ownership. According to § 24(7) S3^37,38^, the state hospital law (LKHG) permits the access to patient data by the contractor if it is ensured by technical protective measures that no personal reference can be established.

In their independence, the federal states are governed by their own hospital laws, which can be restrictive to varying degrees from state to state.

### Aligning GDPR, BDSG and LKHG to implement cloud computing

How can the balancing act between the necessary digitization and the necessary protection of patient data be resolved? We would like to present the approach at a large German university hospital.

Two main methods were implemented to not only comply with German and EU data privacy regulations, but also international requirements: 1) Separation of personal health information (PHI) from medical data and 2) strong encryption of the data, with the storage and access of the encryption keys being restricted to the hospital as data owner or a trusted third party. Allowing a patient to consent or opt-out of the data collection within the platform is a third important aspect. Education and consent of the patients related to the data processing tasks is deferred to the individual hospitals according to their applicable legal constraints. In order to therefore solve the complex interplay of the GDPR with the national and local data sets, consent within the digital ecosystem was characterized as “broad consent” on the basis of § 6(1a) and § 9 (2b) of the GDPR. This grants the possibility for data analysis for various research purposes.

Privacy regulations vary not only internationally, but also within the EU and even within the federal states within Germany. Furthermore, the interpretation of privacy regulation fluctuates and is discussed controversial. Pseudonymized data was considered equivalent to anonymized data if the connection between data and identity cannot be made by a party other than the one possessing the pseudonym-to-identity table^39^. Over the past years, this view changed in a way that anonymized data is only data that cannot be linked to a person by any foreseeable means^40^. With not all medical data in clinical research, even if de-identified, are considered equivalent to anonymized data, patients typically need to be informed of the purposes and all involved parties for processing the data. This is very time-consuming and leads to lower acceptance rate by the patients. Research organizations attempt to generalize the purpose and data processing and exclude any commercial purposes through different types of a “broad consent” in which a patient may agree to data processing for clinical research purposes.

Another way followed by the AIQNET consortium is the clear definition of consented datasets, which are based on legal requirements, such as the collection of data related to the safety and performance of specific groups of and medical devices. The collection and analysis of such data is required by the Medical Device Regulation (MDR)^41,42^. Thus, the MDR serves as legal basis for the collection and processing of clinical data related to the safety and performance of medical devices, according to Art. 6 (1c) of the GDPR^29^.

### Integration of AIQNET at the Charité – University Medicine Berlin

AIQNET, with the Charité as one of its founding members alongside Raylytic GmbH (Leipzig, Sachsen, Deutschland) and BKK B. Braun Aesculap (Melsungen, Hessen, Deutschland) is a consortium consisting of 16 established organizations from various medical-relevant sectors that won the AI competition of the German government in 2019. Work on the infrastructure and applications based on it has been ongoing since January 2020 and is receiving funding from the Federal Ministry of Economics and Climate Protection (BMWi). Currently, it is possible to become a part of AIQNET as an associated partner after careful validation of the consortium. This federally funded project is the first model approach that implements cloud computing for medical AI applications, while establishing means for HIS connectivity and secondary use of medical data for research and compliance purposes as mandated by the MDR.

First and foremost, implementing such an ecosystem in a hospital that is currently still operating with a legacy system and has to maintain its functionality without any capacity for downtimes, is no simple undertaking. By analyzing legacy systems and creating connections in between the systems, AIQNET ensures a step-by-step integration. It processes unstructured and structured information from different medical systems and applications. The connection is established via an integration server that masters the protocols HL7v2, Fast Healthcare and Interoperability Resources (FHIR) and DICOM (Digital Imaging and Communcations in Medicine). This enables the extraction of medically relevant data from legacy systems. As a result, AIQNET supports clinics in automating internal processes.

A migration – or rather integration – of AIQNET involves 1) installation of a virtual machine within the hospital intranet, running the integration server 2) configuration of the transformations between the connected systems and the integration server and 3) configuration of the data collection task (surveys, follow-up time periods, data validation etc.)

**Fig. 1:**
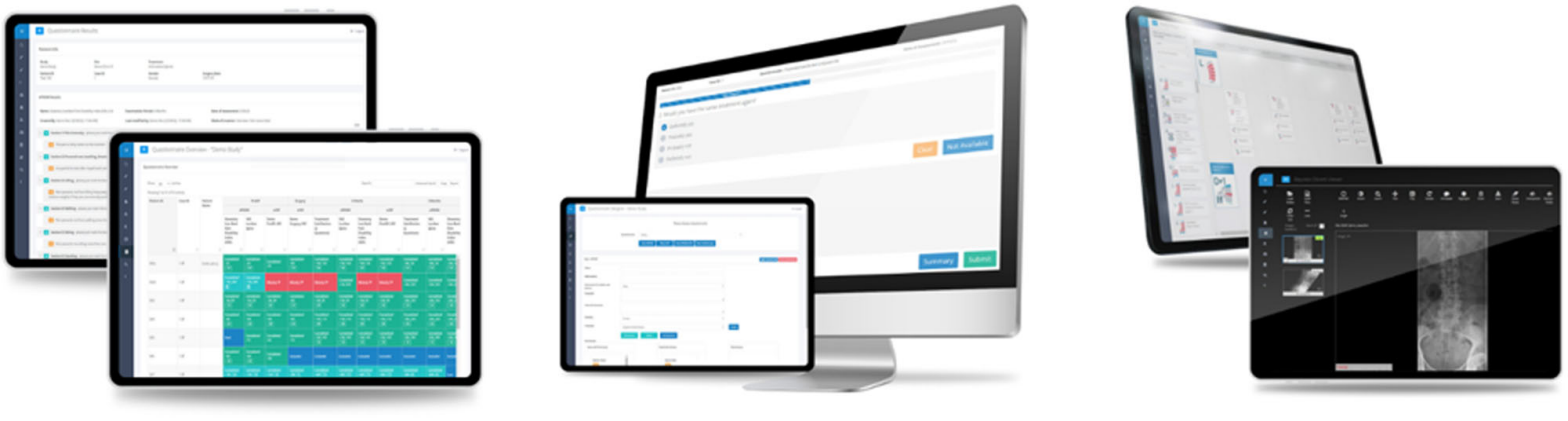
Example of UNITY platform and RayView DICOM viewer.

AIQNET is operated on the UNITY Platform, which represents a granular software-as-a-service module in which various microservices are operated: a DICOM viewer with the option of AI analysis, automated data acquisition, case documentation and outcome recording with patient-reported outcome and experience measurements (PROMs, PREMs). The UNITY platform is developed by the company Raylytic GmBH (Fig. 1) and is the first and only digital solution to do automatic collection of clinical data and AI-powered medical image analysis. The Unity platform is already compliant with GDPR, Google Cloud Platform (GCP) and the Health Insurance Portability and Accountability Act (HIPAA). Furthermore, it is certified according to Information Security Management (ISO) 27001 and ISO 13485. Currently, the UNITY Platform is integrated into the Charité infrastructure for testing purposes. The Spine department of the Charité’s Center for Musculoskeletal Surgery uses it to collect both PROM and PREM data. Also, it has been implemented and prepared for AI analysis of Big Data sets such as pre – and postoperative images of the lumbar spine and whole spine, respectively. The reliability and validity of the AI-analysis software has been proven in prior studies^44^.

The software is characterized by a clear-structured, fine-granular management, which allows only authorized and trained persons access to data with different degrees of access to information regarding patient identity, based on the role assigned. To facilitate this functionality, medical data must be analyzed, de-identified, and linked to a patient via a pseudonym prior to submitting data to the platform. The submission itself occurs through encrypted message protocols, and the storage at rest on the cloud system is encrypted.

The above procedure is exemplified by the upload in the AI-enabled DICOM viewer of RAYLYTIC GmbH. Only authorized staff can upload images via the “RayView DICOM Viewer” after sufficient de-identification. Anonymization and removal of metadata is ensured before upload and storage. An image “fingerprint” reduces replicative uploads of data. Each of these data is further subject to internal quality control to ensure completion of the above requirements and suitability for subsequent analysis processes.

**Fig. 2:**
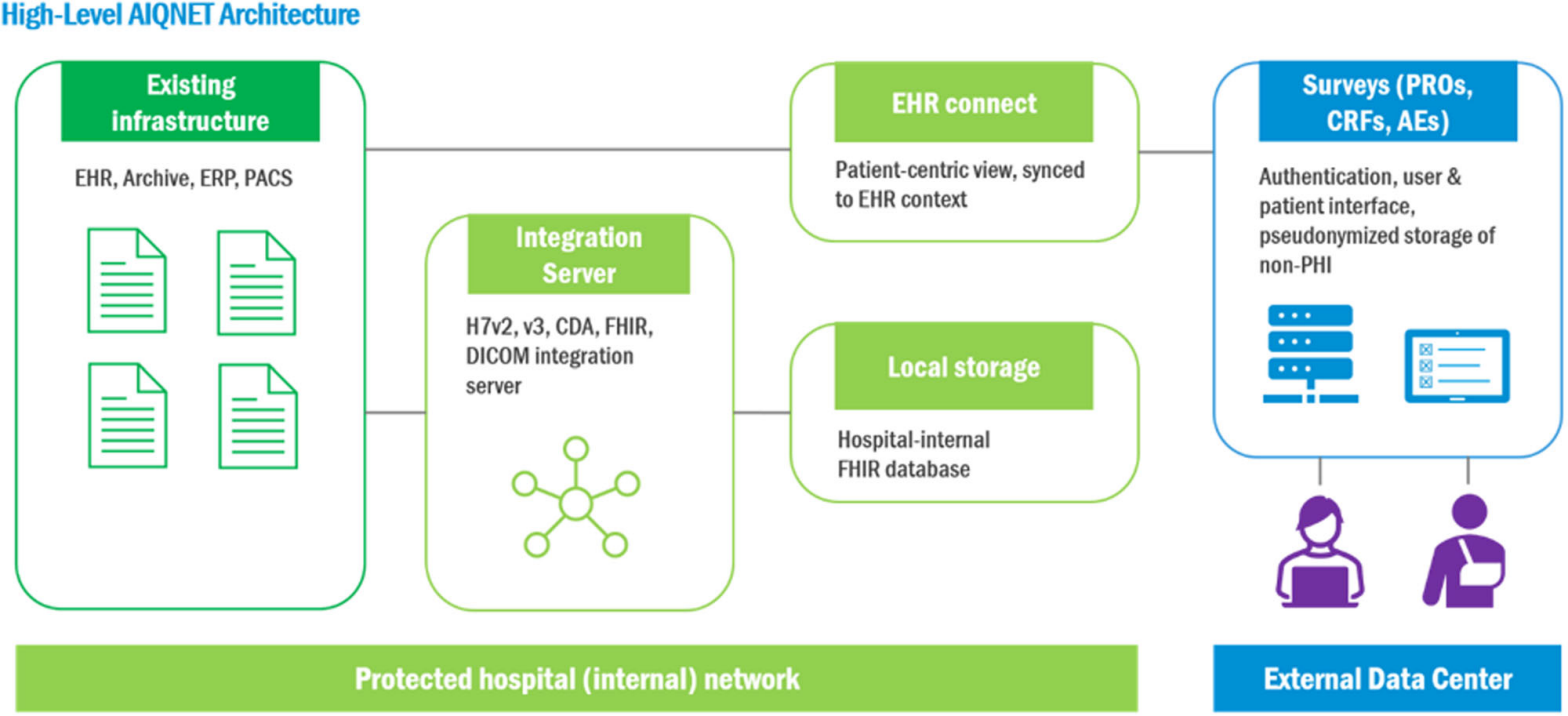
Dataflow and systematic overview of the AIQNET architecture.

The storage of the data takes place in accordance with the GDPR and is securely stored on the local Charité servers. Strict adherence to minimize the storage of personal data is ensured. The design of AIQNET (Fig. 2) foresees 1) PHI never leaving the hospital and 2) only the medical data needed will be collected from the patients or pulled from the Electronic Health Records (HER) systems. Data in transit and at rest is always encrypted. Software architecture, implementation, personnel competence and physical access needed to be independently assessed and a certification according to ISO 27001 was obtained.

In order to ensure that internal quality controls are up-to-date and free of errors, the UNITY Platform and utilized AI algorithms are undergoing regular internal controls regarding the validity of the algorithms and the resulting data integrity.

In view of the legislative requirements, consideration and fulfillment of all these requirements may seem overwhelming in parts. However, within the framework of a funded pilot project, we would like to prove one efficient possibility of implementation and the resulting benefits.

The consortium is focused on establishing interoperability, structuring data with the help of AI and creating a legally secure framework for data-based patient care. In the future, for example, the performance and safety of medical devices can be demonstrated objectively and largely in the safe framework of AIQNET. By contrast, the current legacy implementations of electronic healthcare record systems (EHR) largely prevent the aggregation of data for purposes such as benchmarking or answering highly relevant questions, such as patient outcome associated with a particular type of treatment, device or pathology, and as such, preventing evidence-based precision medicine. Cloud computing, based on an open, standardized data model could help in the transition of hospitals from minimal-interoperable systems to more specialized, interacting services that advance medicine. However, interoperability should not be limited to the local network, but should enable regional, national and, if necessary, international interoperability by means of a coding system commonly used in the consortium – like the Logical Oberservation Identifiers Names and Codes (LOINC) system. With this it is possible to use the UNITY platform of AIQNET, for example, to pool results of Patient-reported outcome measurements (PROMs) by connecting several hospitals within the ecosystem. The UNITY Platform can be used to facilitate Big Data studies, regional and national projects, and prospective, multicenter studies. Data exchange will be further enhanced by the integrated translation of medical and clinical data into a universally exchangeable FHIR format^43,44^.

Cloud computing interfaces that exhibit functionality via an Application Programming Interface (API) empower third parties to access their data programmatically through self-developed or 3^rd^ party applications. A third party is defined as a natural or legal person, public authority, agency or body other than the data subject, controller, processor and persosn who, under the direct authority of the controller or processor, are authorized to process personal data. Examples are data analytics or process automation applications. The AIQNET consortium has streamlined the development of such applications by standardizing on FHIR as data model and “SMART on FHIR” as healthcare IT-systems API definition.

Access of third parties to the data is currently possible via exports of raw data in neutral formats, such as CSV. The exports can be scheduled and transferred via sFTP. In the future, we plan to allow access to the FHIR data through the Smart on FHIR API.

Based on this, AIQNET members are able to develop applications to perform administrative tasks in health care, e.g., to follow-up with a patient, to submit data to a medical registry, to automate the generation of case documentation, or to provide analysis algorithms to aid in treatment decision making. Due to the close cooperation between industry, research and healthcare institutions and the consecutive access to technical and scientific data, the ecosystem’s partners will increasingly benefit for the growing number of applications and medical insight provided through a legally and technically secure, validated framework.

The management of the cloud infrastructure not only ensures a high level of security against the continuously evolving cyber-security risks, but also means that the in-house IT staff can concentrate on user support and infrastructure needs over solving specialist issues related to the maintenance and compliance of legacy systems. Ultimately, by basing the AIQNET ecosystem on open standards and a data model that is receiving a high adoption rate by innovative EHR providers and fairly new players in the healthcare IT space, such as Apple, Amazon Web Services (AWS), Google and Microsoft, the AIQNET participants benefit from long-term investment security of their own development efforts and a growing selection of software applications and human talent.

The close cross-hospital collaboration between pharmaceutical and medical device companies improves the control and monitoring of new products: AIQNET creates an ecosystem for the broad use of health data for research and evidence-based medicine, while complying with legal requirements (compliance). Pharmaceutical and medical device companies also benefit from AIQNET, as they are required by regulatory requirements such as the Medical Device Regulation of the EU (MDR) to continuously monitor their products as part of post-market surveillance (PMS). With AIQNET, hospital data of routine care is generated in a data protection-compliant manner for the testing of the safety and performance of medical devices by notified bodies.

The step towards integration of cloud computing shows the need of cloud computing to keep pace with the rapid development of medicine, the dramatic increase of medical data storage and need for regional, national and international interoperability. Transparent explanations of systematic integration of cloud computing in well-running hospitals are rare and therefore this model approach can be used as a guide. Careful consideration has to be applied when considering privacy regulations within the different member states of the EU, making every case an individual one.

### Reporting summary

Further information on research design is available in the Nature Research Reporting Summary linked to this article.

### Supplementary information

Reporting Summary

## Data Availability

All data relevant to the study are included in the article or uploaded as supplementary information. Data are available on reasonable request.
